# Conditional survival of trimodal therapy for nonmetastatic muscle‐invasive bladder cancer: A SEER database analysis

**DOI:** 10.1002/cam4.4625

**Published:** 2022-03-18

**Authors:** Wentai Shangguan, Jintao Hu, Yingwei Xie, Zhiliang Chen, Qiyu Zhong, Zaosong Zheng, Dingjun Zhu, Yishan Zhang, Jingying Yang, Jinli Han, Wenlian Xie

**Affiliations:** ^1^ Department of Urology, Sun Yat‐sen Memorial Hospital Sun Yat‐sen University Guangzhou Guangdong P. R. China; ^2^ Guangdong Provincial Clinical Research Center for Urological Diseases Guangzhou Guangdong P. R. China; ^3^ Department of Urology, Nanfang Hospital Southern Medical University Guangzhou China

**Keywords:** conditional survival, nonmetastatic muscle‐invasive bladder cancer, prognostic factor, survival analysis, trimodal therapy

## Abstract

**Objective:**

Conventional survival analysis plays a limited role in patients who have survived a period after initial treatment. The present study analyzed how conditional survival (CS) predicted survival rate over time for nonmetastatic muscle‐invasive bladder cancer (MIBC) patients after trimodal treatment.

**Method:**

This retrospective study from the SEER database included consecutive patients with nonmetastatic MIBC who received trimodal therapy (TMT) between January 2010 and December 2017. Kaplan‐Meier analysis was used to estimate overall survival (OS) and cancer‐specific survival (CSS). CS was defined as the rate of surviving y years after already surviving for x years. Multivariate Cox regression analysis was used to identify prognostic factors.

**Result:**

A total of 1110 nonmetastatic MIBC patients treated with TMT were included. Given a 1‐, 2‐, 3‐, and 4‐year after TMT, the rate of surviving to 5‐year, respectively, improved by +5.0 (20.0%), +17.0 (32.0%), +30.0 (45.0%), and +52.8 (67.8%) from those calculated at baseline (15.0%). The 2‐year CS rate of patients who had survived 1‐, 2‐, or 3‐year after TMT improved, respectively, compared to 3‐, 4‐, or 5‐year actual survival. Multivariate Cox regression analysis demonstrated that adverse variables (T stage, age) of OS and CSS lost their prognostic significance over time.

**Discussion and Conclusion:**

Conditional survival rate of surviving to 5‐year after TMT kept a relatively stable level over time. In addition, those adverse variables were not always the prognostic factors over time. Only age was always the significant prognostic factor for conditional OS from baseline to 5‐year survival. Our results provided real‐time survival information and prognosis estimates to adjust follow‐up plans for nonmetastatic MIBC patients after TMT.

## Graphical abstract

Some variables were not always the prognostic factor over time. Only age was always the prognostic factor for conditional overall survival from baseline to 5‐year survival. Our results provided real‐time survival information and prognosis estimates to adjust follow‐up plans for non‐metastatic muscle‐invasive bladder cancer patients after trimodal therapy.

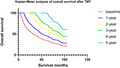

## 1 INTRODUCTION

Bladder cancer (BC) is the seventh most common cancer. There were 81,400 new cases of BC and 17,980 deaths of BC in the United States in 2020.^1^ Approximately 25% of new cases are muscle‐invasive bladder cancer (MIBC).^2^ MIBC is an aggressive disease with a higher probability of metastasis and a worse prognosis.^3^ The 5‐year overall survival (OS) rate for MIBC was only around 5% before the application of radical cystectomy (RC).^2^ Although RC is a usual therapy for MIBC, RC may reduce the quality of life (QOL).^4^ However, trimodal therapy (TMT) with maximal transurethral bladder tumor resection (TURBT), chemotherapy, and radiotherapy has become an effective bladder retention strategy for MIBC.^5^ TMT aims to preserve the bladder without affecting the oncological outcome. The majority of TMT patients achieve a clinical complete response of 60%–80%, avoiding salvage RC.^4^, ^6^ The 5‐year survival rate of the TMT group was not less than that of the RC group, and the efficacy of bladder preservation and QOL were improved.^7^, ^8^ This treatment may become a trend in the treatment of MIBC for selected patients. It is accustomed to using conventional survival analysis like 5‐year survival rate to evaluate the oncological outcome in a static view without considering the changing value of prognostic factors over time.^9^ Therefore, it is necessary to apply better real‐time assessment methods to evaluate the outcome of TMT.

Conditional survival (CS) is recommended to predict the survival time and prognostic factors given the time patients have already survived. The CS is derived from the concept of conditional probability.^10^ CS rate refers to the probability of surviving an additional number of years in patients who have survived a period after initial treatment or diagnosis. CS is likely to be more valuable and more practical than conventional survival analysis because it provides evolving estimates of survival over time.^11^ The application of CS analysis was reported in various malignancies like lung carcinoma,^12^ colorectal carcinoma,^13^ and metastatic renal cell carcinoma.^14^ As for CS on BC, previous studies mainly focused on BC patients treated with RC or neoadjuvant chemotherapy.^15^, ^16^ Our research aimed to explore CS and evolving value of prognostic factors in nonmetastatic muscle‐invasive bladder cancer (NMMIBC) patients after TMT.

## 2 METHOD

### 2.1 Patients and study design

The Surveillance, Epidemiology, and End Results (SEER) database is a public access database and a National Cancer Institute, which covers approximately 28% of the US population.^17^ According to this database, we focused our research on TMT for NMMIBC patients, and patients who met the inclusion criteria were included. Our initial dataset included 148,188 patients diagnosed with BC between January 2010 and December 2017. Exclusion criteria were (1) patients with other primary tumors; (2) patients with unknown information; and (3) patients with distant metastasis and lymph node metastasis. Inclusion criteria were (1) patients who received radiotherapy and chemotherapy after TURBT; (2) T2‐4 BC patients; (3) N0 and M0 BC patients; and (4) exact basic information.

### 2.2 Statistical analysis

Kaplan‐Meier (KM) analysis was used to estimate OS and cancer‐specific survival (CSS). Univariate Cox regression analysis and multivariate Cox regression analysis were conducted to identify the factors associated with survival and screen prognostic factors per year after TMT. Variables, including age, sex, race, histological subtype, grade, and T stage, were incorporated in univariate analysis. Only variables with statistically significant difference (*p* < 0.05) in univariate Cox regression analysis were included in multivariate Cox regression analysis. We used a hazard ratio (HR) with a 95% confidence interval to compare the survival risks of each stratification. CS rate was computed based on KM analysis.

### 2.3 Conditional survival

Conditional survival is the probability of surviving an extra number of y years supposing that the patient has already survived for *x* years. CS can be expressed mathematically as: CS(*x*|*y*) = *S*(*x* + *y*)/*S*(*x*), where *S*(*x*) represents OS rate at *x* years after treatment or diagnosis.^18^ For example, the conditional survival rate (CSR) of the patients who had survived 2 years after TMT and then lived for another 3 years was expressed as CSR (3|2), which is equivalent to 5 years OS rate.

All analyses were conducted using SPSS Version 26.0 and GraphPad Prism 8.0. All statistical tests were 2‐sided with a significance set at *p* < 0.05.

## 3 RESULT

### 3.1 General characteristics of the study population

**FIGURE 1:**
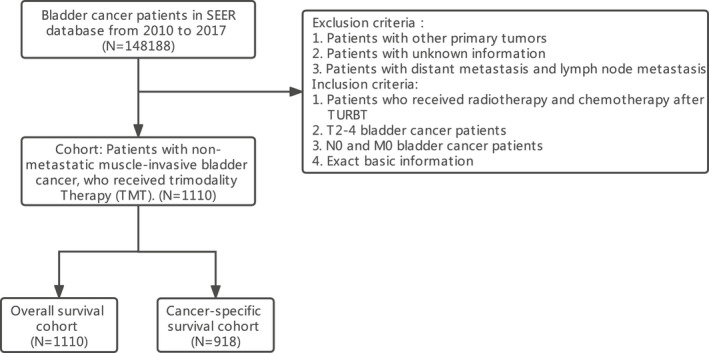
Flow chart of NMMIBC

Among 14,818 patients identified in the SEER from 2010 to 2017, 1110 patients were analyzed after applying the inclusion criteria and exclusion criteria (Figure 1). Overall, 1110 NMMIBC patients, who accepted trimodal treatment, were identified from January 2010 to December 2017. The baseline information was presented in Table 1. There were 833 (75.0%) male cases and 277 (25.0%) female cases. The majority race was white, accounting for 86.8%. Age distribution was as follows: 93 (8.4%) cases were under 60 years old, 236 (21.2%) cases were between 60 and 69 years old, 383 (34.5%) cases were between 70 and 79 years old, and 398 (35.9%) cases were over 80 years old. It could be seen that the majority of all patients were elderly, especially those over 70 years old. There were 944 (85.0%) patients with T2 stage, 92 (8.3%) with T3 stage, and 74 (6.7%) with T4 stage. The majority of all patients were diagnosed with urothelial carcinoma (*n* = 1049, 94.5%). Worse grade (poorly differentiated and undifferentiated) account for 97.2% (*n* = 1079).

**TABLE 1:** Clinical characteristics

Characteristic	All cohort *n* (%)
Sex	
Male	833 (75)
Female	277 (25)
Race	
White	964 (86.8)
Black	89 (8.1)
Other	57 (5.1)
Age	
<60	93 (8.4)
60–69	236 (21.2)
70–79	383 (34.5)
≥80	398 (35.9)
Diagnosis year	
2010	124 (11.2)
2011	102 (9.2)
2012	141 (12.7)
2013	122 (11)
2014	137 (12.3)
2015	156 (14.1)
2016	148 (13.3)
2017	180 (16.2)
T	
T2	944 (85)
T3	92 (8.3)
T4	74 (6.7)
Grade	
Well/moderately differentiated	31 (2.8)
Poorly differentiated	234 (21.1)
Undifferentiated	845 (76.1)
Histology	
Nonurothelium carcinoma	61 (5.5)
Urothelium carcinoma	1049 (94.5)

### 3.2 Actual survival and CS

**FIGURE 2:**
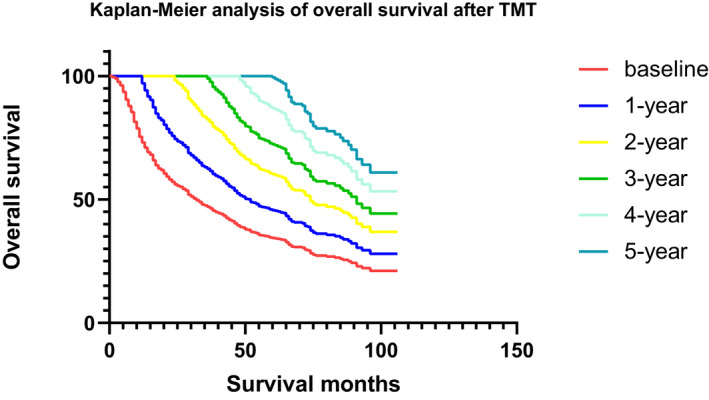
Kaplan‐Meier analysis of overall survival after TMT

**FIGURE 3:**
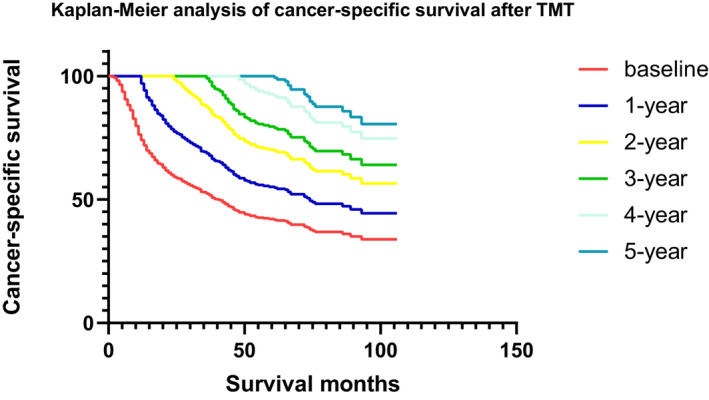
Kaplan‐Meier analysis of cancer‐specific survival after TMT

The median follow‐up of OS and CSS was 22 months. The curve of OS and CSS were presented in Figures 2 and 3. Conditional OS rate (COSR) and conditional CSS rate (CCSSR) of each year increased obviously over time (Tables 2 and 3). Five years OS rate increased from 15.0% directly after TMT to 20.0%, 32.0%, 45.0%, 67.8% given 1, 2, 3, and 4‐year already survived respectively. They can be mathematical as COSR (5|0), COSR (4|1), COSR (3|2), COSR (2|3), and COSR (1|4). CCSSR, CCSSR (5|0), CCSSR (4|1), CCSSR (3|2), CCSSR (2|3), and CCSSR (1|4) were 15.7%, 20.7%, 33.3%, 45.4%, 68.9%. The longer the patients survived, the higher the rate of reaching 5 years of survival. For instance, COSR (3|2) = 45.0% implied that 45.0% of the patients who accepted TMT were alive in the second year would also survive for another 3 years. Furthermore, the 2‐year CSR kept a relatively high level over time for NMMIBC patients treated by TMT. At baseline, the 2‐year actual OS and the 2‐year conditional overall survival (COS) were equal. However, the COS2 had a relatively stable survival rate over time, which was different from the descending actual OS curve. After 1 year of survival, the 2‐year COS was 44.4%, which was improved than the 3‐year actual OS (33.2%). When the patient survived for 3 years, the 2‐year COS reached 45.0% which was significantly higher than the actual 5‐year OS (15.0%). A similar trend was observed in CSS (Table 3). For example, when a patient had survived for 3 years after TMT, the CSSSR (2|3) was 45.4%, which was significantly higher than the actual 5‐year CSS (15.7%).

**TABLE 2:** Conditional overall survival rate

Already survived year	Total years of survival after TMT				
	1	2	3	4	5
0	74.9%	46.8%	33.2%	22.1%	15.0%
1		62.5%	44.4%	29.5%	20.0%
2			71.1%	47.2%	32.0%
3				66.4%	45.0%
4					67.8%

Abbreviation: TMT, trimodal therapy.

**TABLE 3:** Conditional cancer‐specific survival rate

Already survived year	Total years of survival after TMT				
	1	2	3	4	5
0	75.7%	47.2%	34.5%	22.8%	15.7%
1		62.3%	45.6%	30.1%	20.7%
2			73.2%	48.3%	33.3%
3				65.9%	45.4%
4					68.9%

Abbreviation: TMT, trimodal therapy.

### 3.3 Statistical analysis

According to the result of univariate Cox regression analysis at baseline, age, and T stage were significantly correlated to the prognosis for OS (Table 4). Age, T stage, and histological subtype were significantly correlated to the prognosis for CSS (Table 5). Multivariate Cox regression analysis was used to analyze the correlation between variables and prognosis for OS and CSS. Statistically significant variables in the univariate Cox regression analysis were included in multivariate Cox regression analysis. The effect of prognostic factors was estimated by using multivariate Cox regression analysis. HRs were seen in Tables 6 and 7 when compared with the reference. It was testified that T stage and age were significantly correlated to OS and CSS (Tables 6 and 7) at the baseline. Histological subtype lost significant correlation in CSS (Table 7). One year after TMT, age was not associated with CSS. In summary, patients with poorer T stage or older age had a higher survival risk. However, age was the only statistically significant variable 2 years after TMT. The HR of patients over 70 years showed an ascending trend from baseline to 5 years after TMT while the HR of patients between 60 and 69 years old showed a descending HR. The results meant that the survival risk of elder patients may not be associated with disease stage, but older age would significantly affect their prognosis.

**TABLE 4:** Univariate analysis of overall survival

Variables	Subgroups	HR	95.0% CI for HR		*p* value
			Lower	Upper	
Sex	Male	Reference			
	Female	1.077	0.905	1.281	0.403
Race	White	Reference			0.478
	Black	1.177	0.900	1.539	0.234
	Other	1.059	0.752	1.489	0.744
Histology	Nonurothelium carcinoma	Reference			
	Urothelium carcinoma	0.783	0.575	1.067	0.121
Age	<60	Reference			<0.001
	60–69	1.199	0.861	1.669	0.284
	70–79	1.317	0.962	1.802	0.085
	≥80	1.787	1.314	2.432	<0.001
T	T2	Reference			<0.001
	T3	1.263	0.963	1.655	0.091
	T4	1.953	1.485	2.569	<0.001
Grade	Well/moderately differentiated	Reference			0.904
	Poorly differentiated	1.077	0.682	1.703	0.750
	Undifferentiated	1.038	0.670	1.607	0.869

Abbreviations: CI, confidence interval; HR, hazard ratio.

**TABLE 5:** Univariate analysis of cancer‐specific survival

Variables	Subgroups	HR	95.0% CI for HR		*p* value
			Lower	Upper	
Sex	Male	Reference			
	Female	1.150	0.941	1.405	0.172
Race	White	Reference			0.322
	Black	1.260	0.929	1.709	0.137
	Other	0.971	0.632	1.491	0.893
Histology	Nonurothelium carcinoma	Reference			
	Urothelium carcinoma	0.693	0.489	0.983	0.040
Age	<60	Reference			<0.001
	60–69	1.126	0.776	1.633	0.532
	70–79	1.174	0.825	1.671	0.374
	≥80	1.750	1.238	2.473	0.002
T	T2	Reference			<0.001
	T3	1.350	0.994	1.834	0.055
	T4	2.355	1.741	3.187	<0.001
Grade	Well/moderately differentiated	Reference			0.596
	Poorly differentiated	1.046	0.610	1.794	0.870
	Undifferentiated	0.937	0.559	1.572	0.806

Abbreviations: CI, confidence interval; HR, hazard ratio.

**TABLE 6:** Multivariate Cox regression analysis of conditional overall survival from baseline to 5 years after TMT

Variables	Subgroup	Hazard ratio (95% CI) of conditional survival					
		Baseline	1 year	2 years	3 years	4 years	5 years
Sex	Male						
	Female						
Race	White						
	Black						
	Other						
Histology	Nonurothelium carcinoma						
	Urothelium carcinoma						
Age	<60	Reference	Reference	Reference	Reference	Reference	Reference
	60–69	1.224 (0.879–1.705)	1.488 (0.963–2.299)	1.292 (0.735–2.271)	1.165 (0.568–2.392)	1.245 (0.473–3.279)	0.717 (0.192–2.675)
	70–79	1.344 (0.982–1.840)	1.669 (1.102–2.528)	1.483 (0.866–2.538)	1.603 (0.817–3.142)	1.532 (0.602–3.899)	1.671 (0.529–5.282)
	≥80	1.816 (1.335–2.471)	1.993 (1.321–3.006)	2.104 (1.246–3.552)	2.344 (1.220–4.503)	2.946 (1.227–7.072)	2.739 (0.929–8.079)
T	T2	Reference	Reference				
	T3	1.232 (0.940–1.616)	1.134 (0.780–1.648)				
	T4	1.979 (1.505–2.602)	2.108 (1.440–3.087)				
Grade	Well/moderately differentiated						
	Poorly differentiated						
	Undifferentiated						

*Note*: Variables (*p* < 0.05) in univariate Cox regression analysis are included into multivariate Cox regression analysis. Nonsignificant variables (*p* ≥ 0.05) in univariate analysis are blank.

Abbreviations: CI, confidence interval; TMT, trimodal therapy.

**TABLE 7:** Multivariate Cox regression analysis of conditional cancer‐specific survival from baseline to 5 years after TMT

Variables	Subgroup	Hazard ratio (95% CI) of conditional survival					
		Baseline	1 year	2 years	3 years	4 years	5 years
Sex	Male						
	Female						
Race	White						
	Black						
	Other						
Histology	Nonurothelium carcinoma						
	Urothelium carcinoma						
Age	<60	Reference					
	60–69	1.160 (0.799–1.682)					
	70–79	1.203 (0.845–1.713)					
	≥80	1.833 (1.296–2.593)					
T	T2	Reference	Reference				
	T3	1.290 (0.949–1.754)	1.257 (0.815–1.939)				
	T4	2.370 (1.743–3.223)	2.510 (1.580–3.986)				
Grade	Well/moderately differentiated						
	Poorly differentiated						
	Undifferentiated						

*Note*: Variables (*p* < 0.05) in univariate Cox regression analysis are included into multivariate Cox regression analysis. Nonsignificant variables (*p* ≥ 0.05) in univariate analysis are blank.

Abbreviations: CI, confidence interval; TMT, trimodal therapy.

## 4 DISCUSSION

In this study, we researched the CS of NMMIBC patients who were treated by TMT. The COSR and CCSSR of surviving to 5‐year improved from 15.0% and 15.7% at baseline to 67.8% and 68.9% 4 years after TMT. The 2‐year COSR and 2‐year CCSSR are kept relatively stable when compared to the descending trend of actual survival rate. T stage and age were the prognostic factors in OS and CSS at baseline. However, their value of prognosis changed over time. In long‐term OS, the T stage was not always the significant prognostic factor. But age was the only prognostic factor. In long‐term CSS, age lost the prognostic role 1 year after TMT. T stage also lost the prognostic role 2 years after TMT.

In the past decades, RC was considered as the standard treatment for MIBC. However, RC is a challenging operation with significant perioperative and postoperative complications and mortality.^19^ When compared with RC in MIBC patients, TMT presents the huge advantages of preserving the original bladder and guaranteeing the better QOL under the condition that the prognosis of TMT is not worse than that of RC.^7^, ^8^ Although the treatment of MIBC has been improved in the past several years, MIBC is still a malignant disease with 5‐year OS of about 50%.^20^ Therefore, survival assessments are vital for both clinicians and patients in formulating follow‐up strategies and lifestyles.^21^ According to KM analysis, researchers were inclined to get the long‐term outcome of patients by using cumulative survival rate, without taking the changing factors into account.^15^ The OS rate for NMMIBC patients with TMT was even 15.0% in our study. Poor prognosis meant a loss of more cases. The research background, patients' basic information and disease situation had transformed into different conditions over time. Therefore, conventional cumulative survival analysis could not satisfy our requirements of survival analysis and reflect the real‐time background in survival rate over time after preliminary survival analysis, particularly when patients with poor prognosis had survived several years. However, CS, a dynamic survival calculation method, reflects the changes in survival rate and provides real‐time survival prognostic factors with the increase in follow‐up time.^18^ CS then applies to patients with cancer, especially those with poor prognosis such as lung,^12^ colon,^22^ and brain^23^ cancer. In our research, the survival rate of NMMIBC after TMT was poor. Therefore, it was more helpful to apply CS to our study, which was in favor of clinicians to adjust follow‐up visits plans and enhance the survival confidence of patients.

In the present study, the rate of achieving 5‐year OS and CSS of NMMIBC patients treated with TMT improved obviously as survival time increased. Similar improvement was shown in other malignancies.^12^, ^21^ The 2‐year COS and the 2‐year conditional CSS well improved over time when compared with the actual survival rate. For instance, the actual 5‐year OS rate was 15.0%. However, the rate of surviving to 5 years was improved to 45.0% after 3 years of survival. CS seemed to provide inspiring follow‐up information. In addition, we found that histological subtype was not a significant variable in the multivariate Cox regression analysis of CSS. But, T stage and age were statistically significant at the baseline in OS and CSS. Poorer T stage or elder patients had greater HR than the reference. One year after TMT, the T stage and age were still associated with the prognosis of OS, but age was no longer associated with the prognosis of CSS. Interestingly, only age became a statistically significant prognostic factor for 2–5 years OS after TMT. However, there were no statistically significant prognostic factors for 2–5 years CSS.

Kang et al. also demonstrated that age was the only significant prognostic factor in 5 years OS for MIBC patients who were treated by RC. Stage and grade also lost their value of prognosis.^15^ Palumbo et al. demonstrated CS provides obvious survival gains for patients who received RC. T stage and N stage were significantly related to the survival prognosis and manifested the survival gain of CS as well. However, the T stage and N stage lost their statistical significance 5 years after surgery in CSS. They did not research the effect of age. In our study, CS also provides obvious survival gains for patients who received TMT. Two years after TMT, age was considered as the only significant prognostic factor for OS. T stage lost the statistical significance 2 years after TMT for OS and CSS.^24^ Sun et al. reported that survival for the initial 2 years after RC was vital to the subsequent prognosis of patients.^25^ Ritch et al. built a model to demonstrate that TMT resulted in a relatively low mortality at 1 year. However, there was a significant and persistent higher mortality 2 years after TMT. Patients treated with TMT had multiple comorbidities.^26^ Comorbidities may explain the results in the present study. Older age meant a higher probability of having comorbidities and higher risk of dying from comorbidities, not tumor‐related factors. Therefore, CS was not only significant 1 year after TMT but also necessary 2 years after TMT for NMMIBC patients. T stage could not be regarded as the forever prognostic factor. A poorer stage may get better oncological outcome. The worse prognosis of TMT over time was destined to update the latest prognostic information.

Although grade variable was used to be regarded as the prognostic factor of BC, few cases of well‐differentiated or moderately differentiated may form statistical deviation in our study. Usually, for some patients with poor prognosis, age, gender, disease stage, and histological type may all affect prognosis at the beginning of disease diagnosis. However, as time goes by, the prognostic factors for patients' survival may only be age or some other factors, or even no statistically significant prognostic factors. It can no longer be considered that stage, histological type, and grade of disease will always affect the survival time of patients. Especially 2 years after TMT in our study, T stage was not associated with OS estimate. When compared with patients younger than 60 years old, older patients had greater HR value. And patients over 70 years had increasing HR from baseline to 5 years after TMT. But there was a decreasing HR for patients between 60 and 69 years old. It demonstrated that elderly patients had higher risk of survival and age was the only variable in the long term of 5‐year OS. Besides, no variables are always significantly correlated to the estimate of CSS.

In summary, our study was the first study to research the CS of TMT. And we found survival gains in the CS of TMT. Those adverse prognostic factors at diagnosis would lose their significance over time. Finally, age became the only prognostic factor 1 year after TMT. Our research provided more confidence and information for those patients with worse disease stage who had survived 1 year after TMT. These data can also allow clinicians to better assess patients' survival conditions and prognostic factors. Our study also provided valuable information to guide real‐time follow‐up plans for patients with NMMIBC after TMT. Despite our research having the above advantages, there are some limitations in our research. Firstly, the research was a retrospective cohort with a limited number of patients and higher selection biases. Secondly, we could not get the latest grade data from the SEER database. Thirdly, due to few numbers of patients with the well‐differentiated grade or the moderately differentiated grade, we integrated those patients into one group for CS calculations, which may cause biases for identifying more accurate prognostic factors. Therefore, survival months may not represent the OS and CSS of patients with a better grade. Fourthly, we could not include some other vital information from the SEER database, such as concrete comorbidities, BMI, smoking history, radiation exposure history, recurrence, and the detailed treatment records of surgery, radiotherapy, and chemotherapy. Fifthly, the follow‐up months of the SEER database was based on the time after diagnosis, not the time after treatment. Survival estimate may not be exact and may cause biases.

## 5 CONCLUSION

In general, COS and CSS analysis provided a different view on survival rate over time in patients with NMMIBC after TMT compared with conventional survival analysis. Particularly, CS demonstrated a stable survival rate and obvious survival gains over time in our study. In addition, some acknowledged prognostic factors, including T stage and grade, were not significant anymore after a short term of follow‐up. Interestingly, from baseline to 5‐year survival, only age was always the significant prognostic factor for COS. The research offered valuable information to guide real‐time follow‐up plans for patients with NMMIBC after TMT.

## CONFLICT OF INTEREST

The authors declare that the research was conducted in the absence of any commercial or financial relationships that could be construed as a potential conflict of interest.

## AUTHOR CONTRIBUTIONS

Conceptualization was performed by Wentai Shangguan and Wenlian Xie; Data curation was performed by Wentai Shangguan; Formal analysis was conducted by Wentai Shangguan, Yingwei Xie, Yishan Zhang, and Jingying Yang; Funding acquisition was done by Wenlian Xie; Methodology was performed by Wentai Shangguan, Jintao Hu, and Zaosong Zheng; Software was by Wentai Shangguan and Jintao Hu; Supervision was performed by Wenlian Xie; Validation was performed by Yingwei Xie, Zhiliang Chen, and Qiyu Zhong; Writing—original draft was done by Wentai Shangguan and Jintao Hu; Writing—review and editing was done by Yingwei Xie, Jinli Han, Dingjun Zhu, and Wenlian Xie. All authors have read and agreed to the published version of the manuscript.

## ETHICAL APPROVAL STATEMENT

All data analyzed in this study are publicly available from the Surveillance, Epidemiology, and End Results (SEER) database (https://seer.cancer.gov/). No ethical approval and patient consent are required.

## Data Availability

The data and materials analyzed in the current study are available from the corresponding author on reasonable request.
